# Study on the Restoration of a Masonry Arch Viaduct: Numerical Analysis and Lab Tests

**DOI:** 10.3390/ma13081846

**Published:** 2020-04-14

**Authors:** Damian Beben, Janusz Ukleja, Tomasz Maleska, Wojciech Anigacz

**Affiliations:** Faculty of Civil Engineering and Architecture, Opole University of Technology, 45-758 Opole, Poland; j.ukleja@po.edu.pl (J.U.); t.maleska@po.edu.pl (T.M.); w.anigacz@po.edu.pl (W.A.)

**Keywords:** masonry arch viaduct, numerical analysis, bridge load, restoration

## Abstract

This article presents an analysis of the load-carrying capacity of a historic masonry arch viaduct. The vault was made of bricks and lime-cement mortar. It was built in 1886 and, therefore, its historical character had to be included in the restoration project. The main task of the restoration was to bring the viaduct to a technical condition corresponding to the current requirements to allow normal (or limited) service. The strength of the brickwork and joints (mortar) was examined experimentally in the laboratory and on the viaduct. This paper presents numerical calculations for the masonry viaduct that were performed using two programs based on the finite element method. As the project documentation was unknown, two- and three-hinged models of the masonry arch were analyzed. The axial forces, shear forces, bending moments, displacement, normal stresses, and shear stresses generated from the numerical analysis have been discussed. The conditions of the load capacity of the arch viaduct due to compression and shearing have been met. The safety of a masonry arch of the viaduct was determined. Finally, the restoration scope of the masonry viaduct was proposed.

## 1. Introduction

Currently, numerous modernizations of public roads and railway lines are being carried out to adapt these routes to new terms of use, mainly in terms of increased service loads. For this reason, many bridges located on these routes also require immediate restoration and/or reinforcement. The main reason for this is the poor technical condition of bridges and viaducts [1,2,3]. Restored bridge structures must meet the appropriate requirements [4,5]. In addition, a large proportion of these bridges are historic objects protected by several applicable conservation regulations [6].

The structural response of masonry arch bridges is strongly related to the arch geometry, the brick (or stone) dimensions, the quality of the mortar, and the interaction phenomenon between the arch and backfill [7,8,9,10,11]. Experimental tests and numerical analyses of masonry arch structures have been repeatedly executed. The load-carrying capacity of masonry bridges was analyzed using the advanced discrete element method [12,13]. Besides, an implicit discrete element method was used to analyze the mechanical behavior of a standard arch bridge and a masonry stone bridge [14]. In some cases, to predict the nonlinear structural behavior of masonry arches, a new discrete macro-element method was applied [15]. A novel approach was proposed for studying masonry arch bridges using inverse analysis procedures [16,17]. The authors adopted a global optimization approach using a genetic algorithm. A Bayesian approach was also used. Nonlinear 3D finite element (FE) models, including arch-fill interaction effects and limit analysis models, were also developed [10,18,19,20,21,22,23,24]. The arch in masonry arch bridges was analyzed as a two-phase material comprising bricks (or stones) and mortar [13,25]. An accurate mesoscale arch masonry model was also proposed [26,27,28,29,30]. The nonlinear response of brick-masonry arches was studied, including nonlinear interface elements for describing mortar joints and brick-mortar interfaces. 

The authors of a study [31] presenting an orthotropic damage model of a multi-span masonry railway bridge for structural analysis described the mechanical behavior of masonry using an original 3D anisotropic damage model that could consider the opening and the progressive reclosing of localized cracks. The model also used homogenized parameters for the weakness of the stone-mortar interface. Another study investigated the influence of the elastic modulus on stone arches through micro-modelling (including defining the scope of the elastic modulus in the failure mode and collapse load) using the FE method [32]. Results from the uniform and multi-point response spectrum analyses cases using a 3D FE analysis of the effects of ground shocks due to explosive loads in historical masonry bridges showed that there were significant differences between the uniform and multi-point blast-induced ground motions [33]. 

A numerical model of the masonry arch railway bridge was calibrated using dynamic modal parameters estimated from ambient vibration [34]. The problem of flood-induced scour on masonry arch bridges was also investigated using nonlinear three-dimensional modelling [29,35].

Typically, masonry arch bridges need restoration and should be effectively strengthened while maintaining historical value. Traditional techniques of strengthening (steel bars, stirrups, steel profiles, injecting cementitious mortar, reinforced concrete hoods, and introducing ties at the arch impost) are being abandoned due to the aesthetic incompatibility and extra self-weight and rigidity that these techniques could add to the structure [36]. Recently, composite materials have been used instead of traditional techniques, because they are considered innovative (carbon fiber reinforced polymer, glass reinforced polymer, steel rod, steel-reinforced grout, basalt textile-reinforced mortar, or other fiber materials) [37,38,39,40,41,42]. An interesting way of reinforcing a masonry arch railway bridge was recently presented [43]. Polyurethane polymer was applied to study the stabilizing effects on the bridge. Altunisik et al. [23] investigated the effect of restoration on the structural behavior of masonry arch bridges. The structural responses of the bridge were obtained before and after the restoration under dead, live, and dynamic earthquake loads.

To determine the actual properties of masonry arch bridge components, it is necessary to conduct the appropriate test procedure. Non-destructive, minor-destructive, and monitoring methods are used to assess masonry arch bridges [22,34,44]. A new assessment method to identify the historic masonry arch bridges was also proposed [45]. The deformed bridge geometry was quantified using laser scanning technology and point cloud processing. Zheng et al. [46] also analyzed the impact of acid rain on brick properties. 

As can be seen from the above, the analysis of masonry arch bridges has been undertaken for different aspects and different methods of analysis. Usually, an advanced numerical analysis was performed for this purpose, using available calculation programs. However, from a practical point of view, the bridge engineers usually use a relatively simple calculation approach to assess the load-carrying capacity of the bridge. In many cases, they do not have advanced calculation tools and test capabilities, which is why it is important to know what calculation error occurs with simple bridge modelling.

The subject of the article is a masonry arch viaduct that has undergone restoration while maintaining its historic character. First, the technical conditions (in the laboratory and in-situ) of individual viaduct elements were examined, i.e., the arch structure, wings, and headwalls. On this basis, the scope of the necessary restoration work was determined. A static-strength analysis of the masonry arch viaduct was performed using relevant bridge standards. Its aim was to determine whether the masonry arch of the viaduct will tend to lose load capacity for the anticipated railway load for a 40 km/h train speed. This article presents two FE methods to determine the load-carrying capacity of the masonry arch viaduct. The first used Autodesk Robot Structural Analysis software [47], named program #1 in this paper. This is an engineering program used to calculate relatively simple tasks. The second calculation method was conducted using DIANA [48], named program #2. This is a more advanced software that can take into account, for example, various soil models and the interaction between the backfill and masonry arch. This paper answers the question of whether the analysis of the masonry viaduct can be conducted using simple engineering models without significantly increasing internal forces. Additionally, this paper includes an analysis of various static schemes of the arch (two- and three-hinged arch). A two-hinged arch corresponds to the actual static scheme. In addition, a third hinge located at the crown was used because this place was damaged by being hit by a non-standard vehicle and may be exposed to hinge formation. The conclusions relate mainly to the viaduct arch safety, the possibility to use simple modelling, and the influence of the arch static scheme. The proposed method of analysis and restoration can be used for similar masonry arch bridges. 

## 2. Viaduct Description 

The masonry arch viaduct was built in the late nineteenth century. Its last renovation was carried out in 1983. The load-carrying structure of the viaduct is a brick arch structure in a semicircle shape with a vault thickness of about 0.50 m. The vault is made of bricks on lime-cement mortar. The span is 6.00 m, the vertical clearance is 4.26 m, and the total width is 7.42 m. The backfill height over the arch crown is 0.63 m. The backfill was made of sandy soil with a 36° internal friction angle, density index *I_D_* = 1.0, and 22.8 kN/m^3^ unit weight (density). The load-carrying capacity of the object before restoration was determined as for railway bridges with limited train speed. There was a speed limit of 20 km/h for trains. It was not possible to identify the exact shape of the arch viaduct structure from the ground side in the support zone and its foundations (there is no original project documentation). It was, therefore, assumed that the structure of the arch was probably founded directly on the ground. To perform numerical calculations, it was necessary to perform an inventory and analysis of the documentation of other similar viaducts built at that time, using analogies to the shape and construction of these objects.

**Figure 1:**
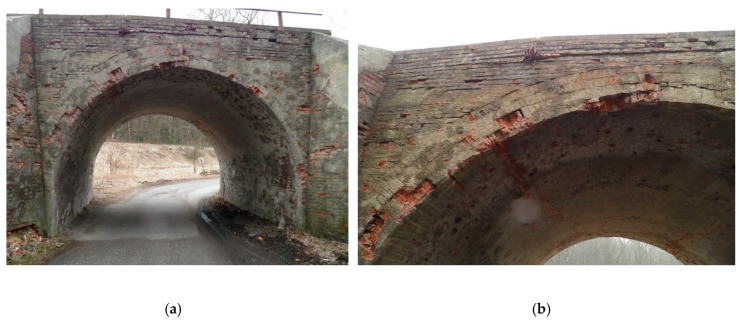
Side view of masonry arch viaduct before restoration: (**a**) Overview, (**b**) visible damage (deflection from the vertical) of the parapet (upper part of the headwall), which was caused by the root.

At the inlet and outlet of the viaduct, there were brick wings with a variable thickness of 0.60–1.20 m and a length of about 4.50 m. The lack of ongoing maintenance and regular repair works to the viaduct over the past 30 years has led to its poor technical condition, mainly in the field of the aesthetic perception (Figure 1a). 

During the research, it was established that the technical condition of the viaduct arch was sufficient. During the inspection, it was found that there was superficial damage to the carrying masonry arch, but it did not show a risk of stability loss. Damage and cracks in the headwalls and numerous defects in joints were also noted. Some bricks were also missing. The condition of the headwall was rated as satisfactory. A root was noted growing on the parapet (top part of the headwall), which caused its slight local deviation (<5 cm) from the vertical position (Figure 1a,b). The deviation was located in the upper part of the headwall (outside the area of backfill pressure on the headwall) and did not affect the load-carrying capacity of the masonry arch. There was weeping and blooming from the bottom of the span, which indicates insulation leakage. In addition, leaching of the joints between bricks and the brick surface destruction were visible. However, in general, the lime-cement mortar was in fairly good condition. Superficial damage to the masonry arch structure caused by impacts from non-standard vehicles was noted. The insulation condition of the viaduct was bad; the drainage was in a slightly better condition.

## 3. Laboratory Testing of Materials

Determining the actual load-carrying capacity of the masonry arch viaduct requires knowledge of the real strength of the bricks and mortar. For this purpose, it was necessary to check the technical condition of the bricks. A dozen bricks were taken from the viaduct and part of the wall for detailed studies in the laboratory. The technical condition of the bricks was quite good (bearing in mind the long lifecycle of the viaduct). The results of the laboratory tests on the viaduct materials were as follows:

The compressive strength of the bricks was tested using a Marshall Test press according to PN-EN 772-1:2001 [49]. Sixteen samples (40 × 40 × 80 mm) cut from the bricks derived from various parts of the arch were used for testing (Table 1). Tests were carried out after the samples were dried to a constant weight (eight samples), and in a state of water saturation (eight samples) due to the high degree of moisture in the components of a brick wall. The compressive strength of the bricks dried to a constant weight was in the 15.62–32.87 MPa range (mean 23.31 MPa) and for samples soaked with water, it was 7.93–25.37 MPa (mean 16.29 MPa). The coefficients of variation for the compressive strength of bricks dried to constant mass and soaked with water were 24.64% and 34.27%, respectively (Table 1). These coefficients seem to be quite large; however, taking into consideration the age of the viaduct (over 100 years), they are justified. In addition, the tested bricks came from different parts of the arch (crown, haunch, the lower part of the arch). This proves that bricks in the arch were decayed to varying degrees, which ultimately translated to their different strength. 

Tests for the compressive strength of the wall samples (and, indirectly, the mortar) were carried out in the laboratory using a Marshall Test press per PN-EN 1052-1:2000 [50]. Additionally, compressive strength tests were carried out on the viaduct (in-situ test) using the sclerometric method (Schmidt hammer). Fourteen samples (seven in the lab—derived from the viaduct arch—and another seven in-situ) were tested (Table 2). The compressive strength of the brick wall in the laboratory was in the 12.9–18.2 MPa range (mean 15.6 MPa), and when tested in-situ, 14.8–21.9 MPa (mean 17.3 MPa). Table 2 also shows that the coefficients of variation did not exceed 15%. In addition, the compressive strength of the lime-cement mortar was assumed as 10 MPa. 

The shear strength of seven wall samples (and, indirectly, the mortar) were investigated in the laboratory per PN-EN 1052-3:2004 [51]. Table 2 presents the results and they were in the 0.36–0.40 MPa range (mean 0.38 MPa). The coefficient of variation did not exceed 4%.

The weight absorbability study was carried out per PN-EN 13755:2008 [52]. The study was performed on material samples taken from the viaduct. The necessity for conducting these tests was due to the high level of moisture in the brick wall elements. The average weight of the brick samples dried to a constant mass and of bricks soaked with water was 220.69 and 245.82 g, respectively. The coefficient of variation did not exceed 5.5% in both cases. The values of the weight of water absorption of the ceramic material samples were in the 7.1–13.6% range (mean 11.39%, the coefficient of variation did not exceed 19%). 

## 4. Analysis of the Masonry Arch Viaduct

### 4.1. General Notes

The moving loads of railway bridges were adopted per PN-85/S-10030 [53] and PN-EN-1991-2 [54]. When checking the load capacity of the railway viaduct, the following standard moving loads were taken into account: (i) Load with rolling stock, (ii) load caused by braking and acceleration, (iii) load caused by side impacts of rolling stock. 

Due to placing the track on the ballast with a thickness in the range of 0.50 m < *h* = 0.63 m ≤ 1.0 m from the top of the sleeper, the calculated dynamic amplification factor (as for a carefully maintained track) was equal to 1.53. With regard to bridges with a program-limited speed of 20 km/h (the railway administration has imposed a condition for obtaining the possibility of train movement at 40 km/h), the dynamic amplification factor was reduced, and in the considered cases, it amounted to 1.08 for 20 km/h and 1.23 for 40 km/h. The input values for the calculations were determined, i.e., axle load pressure *P* = 200 kN and uniformly distributed load *P* = 72 kN/m. Therefore, the values of *P* = 72 kN/m and *Q* = 200 × 4/6.4 = 125 kN/m were accepted for calculations. Ultimately, the loads with rolling stock were *Q_t_* = 202.5 kN/m and *q_t_* = 116.6 kN/m. 

The load caused by braking forces was assumed as horizontal forces amounting to 1/10 of the load without load length limitation per [49], and it, therefore, amounted to *E_bra_.* = 229.2 kN and *q_T_* = 165.8 kN. Meanwhile, the acceleration forces were assumed as horizontal forces amounting to 1/5 of the load with forces *P* and they were equal to *q_acc._* = 208 kN. Finally, the acceleration force was adopted as more unfavorable. 

The simplified calculation model (in program #1) adopted the basic forms of loads (dead weight of the arch, soil weight and subgrade, load with railway rolling stock, braking and acceleration, side-impact, and active earth pressure). Additionally, six load combinations taking into account the worst setting and different values of the coefficients were considered (Table 3). 

In program #2, the applied loads were generally almost the same as in program #1. The differences were related to backfill modelling in both programs. Program #2 uses 3D elements, so the active earth pressure (*F*) was included automatically. Taking into account the most unfavorable load conditions, three load combinations (4–6) were adopted in both applied programs and compared to each other. 

### 4.2. Strength Parameters of Brick Wall

The results obtained from the material tests (Section 3) were used to calculate the current load-carrying capacity of the analyzed masonry viaduct. The design compressive strength of the masonry wall was calculated per PN-B-03002 [55] and it was 3.20 MPa. The design shear strength of the masonry wall was determined per [51] and it was 0.51 MPa. The modulus of elasticity of the masonry wall amounted to 3246 MPa. Poisson’s ratio for a clinker brick wall was equal to 0.2. Kirchhoff’s ratio was also calculated, and it amounted to 1.360 MPa. Specific gravity was adopted according to PN-82/B-02001 [56] as for clinker brick and it was equal to 19 kN/m^3^. The thermal expansion coefficient was also assumed (6 × 10^−6^ 1/°C) as for clinker brick.

### 4.3. Description of the Calculation Models

**Figure 2:**
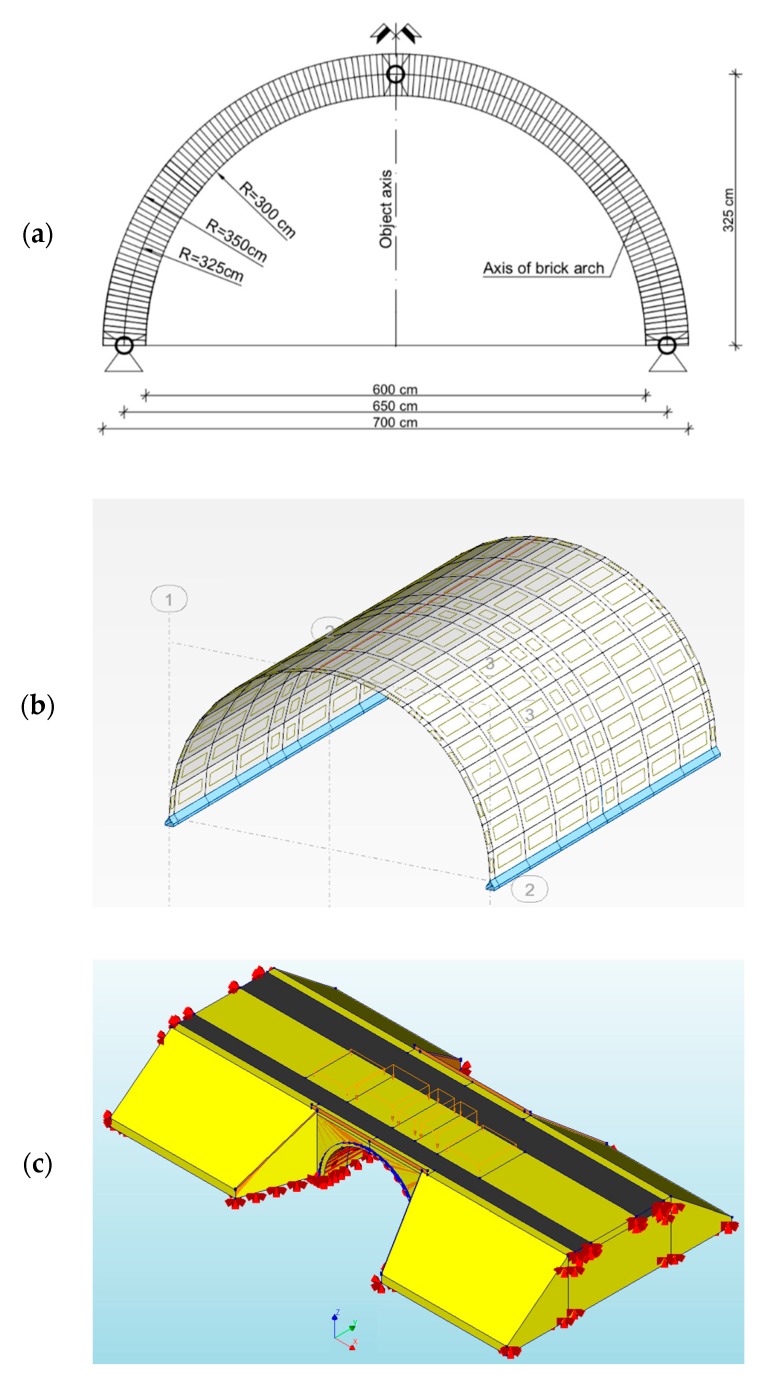
Viaduct with three-hinged arch: (**a**) Static scheme, (**b**) program #1, (**c**) program #2.

The brick wall on lime-cement mortar creating an arch was made continuously (without expansion joints and hinges). However, in the calculations, the authors assumed that, in unfavorable circumstances, transverse cracks might occur. This assumption was due to potential internal structural damage of the arch (joints). In the analysis, two models of an arch were considered, i.e., two- and three-hinged (Figure 2a). According to preliminary calculations using the two-hinged arch, permissible normal stresses from the standard load were not exceeded (Table 4). However, given the technical condition of the viaduct (damage to the arch crown resulting from being hit by a non-standard vehicle), there was the possibility of creating a third hinge in the crown. 

The arch in the shape of a cylindrical shell with a span edge in its crown and two-hinged supports on its lower edges was subjected to analysis (Figure 2b). Additionally, in the next numerical model, an additional hinge was introduced at the arch crown, forming a three-hinged arch (Figure 2a). The shell structure was modelled with a constant 0.5 m thickness and strength parameters of the brick wall and curvature consistent with the neutral axis of the actual arch. In program #1, the arch shell structure was divided into 2369 FE of rectangular shape, while in program #2, the arch had 2580 FE, but in the whole numerical model, there were 243251 FE. 

The mechanical parameters of the brick wall were adopted based on experimental (lab) tests and calculations per PN-B-03002 [55]. The brick wall on lime-cement mortar was loaded with a structure dead weight amounting to 18.0 kN/m^3^. The backfill modelling in program #1 and #2 was in two different approaches. In program #1, the backfill above the arch was assumed as the soil weight with the given height. Meanwhile, in program #2, the backfill was modelled as a 3D model using the Duncan‒Chang nonlinear elastic hyperbolic model [57]. The backfill was modelled using solid elements (HX24L) with the failure ratio *R_f_* = 0.7, unloading‒reloading stiffness *E_ur_* = 1000 N/m^2^, reference pressure *P_ref_* = 101,350 N/m^2^, exponent for unloading reloading curve *m* = 0.25, exponent for backbone curve *n* = 1.1, 350 N/m^2^ minimum compressive stress, 5° dilation angle, and 3 kPa cohesion [58]. Based on the embankment outcrops investigated, it was assumed that the backfill was made of sandy soil with a 36° internal friction angle, density index *I_D_* = 1.0, 22.8 kN/m^3^ unit weight, 100 MPa Young’s modulus, and Poisson’s ratio of 0.2. The unit weight of the ballast (broken stone—being a foundation for railway tracks) was assumed at 21.6 kN/m^3^.

The soil-structure interaction phenomenon between the masonry arch and backfill was modelled using a special function in program #2. The properties of the interface were a “Coulomb friction” function with a 36° internal friction angle, 3 kPa cohesion, 5° dilation angle, and 1,000,000 kN/m^3^ rigidity [59]. Meanwhile, in program #1, the backfill was only the load, without interaction between the soil and the arch structure. In program #1, the unit active earth pressure at 0.5 m was 1.5 kN/m^2^ and it was 9.0 kN/m^2^ at 3.0 m below the soil level. 

### 4.4. Numerical Analysis of Bridge Arch

#### 4.4.1. General Remarks

Two computational programs were used in the numerical analysis. Program #1 is a tool designed mainly for engineers, where it is possible to determine the values of maximum displacements, stresses, axial forces, or bending moments simply and easily. Drawbacks of this software are the limited way of modelling the entire model and the poor range of available material models. In addition, the limitations in the numerical modelling included the following assumptions:

- an arch wall with properties corresponding to clinker brick has material continuity,
- the possibility of spontaneously creating linear hinges in the wall (enabling rotation and without the possibility of sliding) in vertical arch supports,
- the curvature of the arch was modelled in the form of flat panels divided into flat FE,
- no interaction between backfill and arch structure,
- the external forces were projected on the axis of the panels, not applied to their surface.

**Figure 3:**
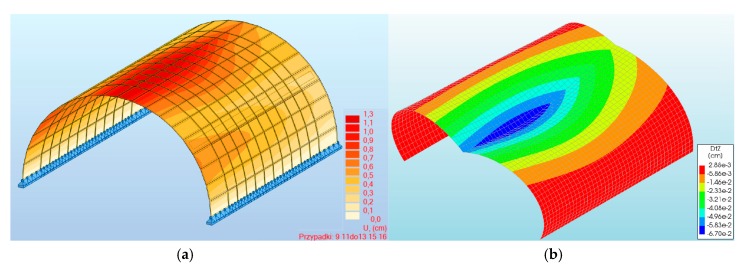
Maximal displacements of the three-hinged arch for program: (**a**) #1, (**b**) #2.

**Figure 4:**
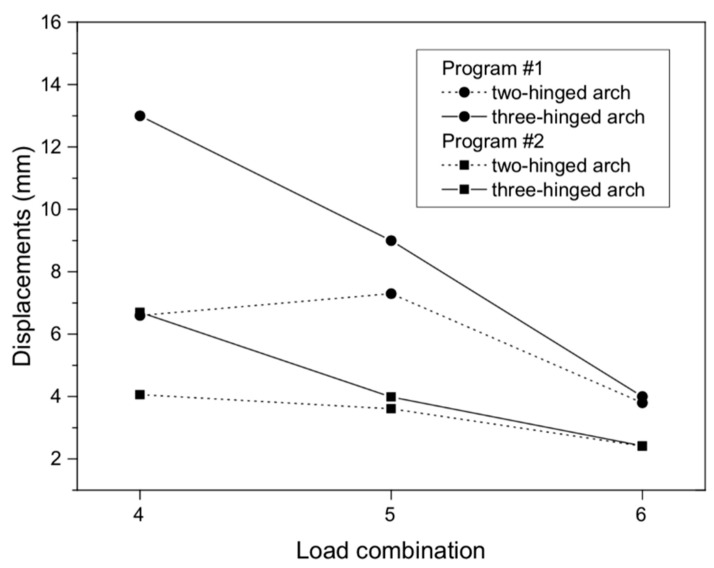
Displacements of masonry arch for various load combinations.

**Figure 5:**
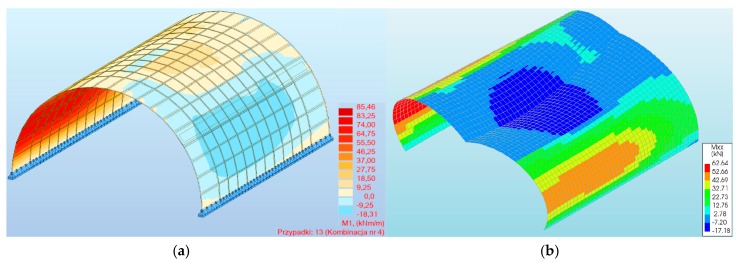
Maximum bending moments of a three-hinged arch for program: (**a**) #1, (**b**) #2.

**Figure 6:**
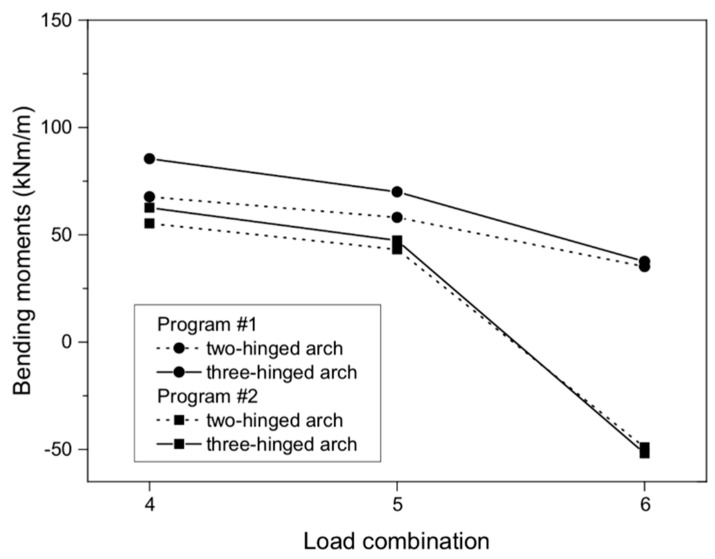
Bending moments of masonry arch viaduct for load combinations.

**Figure 7:**
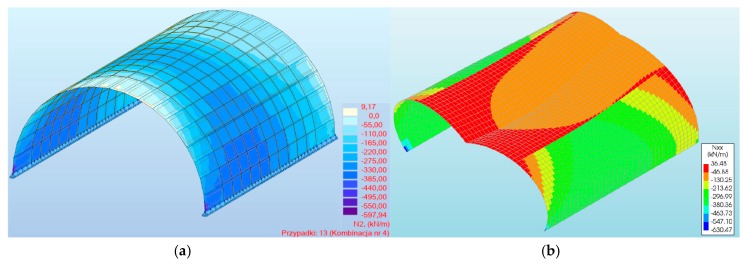
Maximum axial forces of a three-hinged arch obtained in the program: (**a**) #1, (**b**) #2.

**Figure 8:**
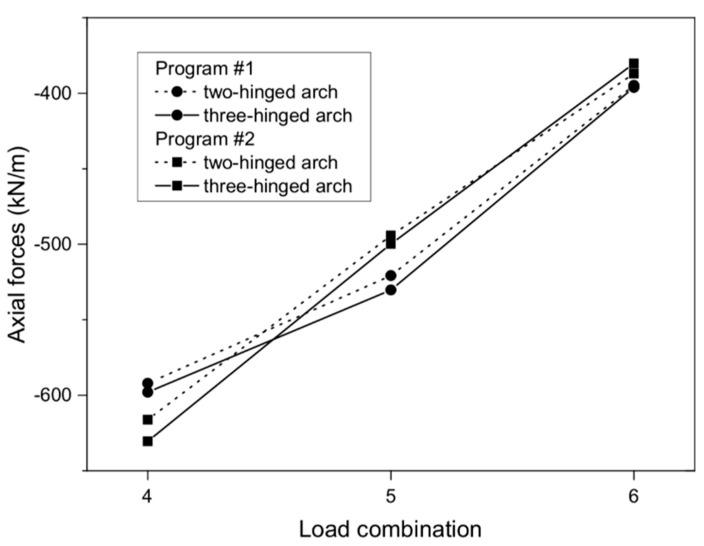
Axial forces of arch viaduct vs. load combinations.

**Figure 9:**
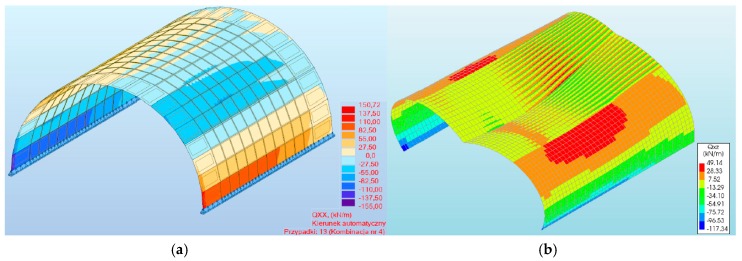
Maximum shearing forces of a three-hinged arch viaduct obtained in the program: (**a**) #1, (**b**) #2.

**Figure 10:**
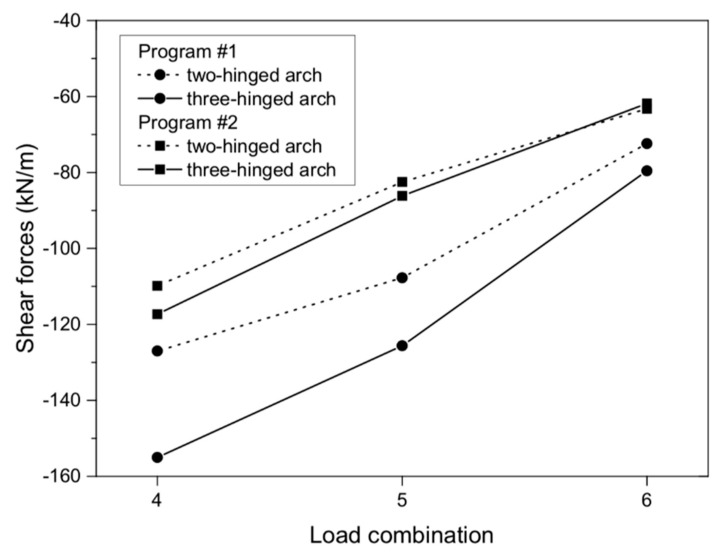
Shearing forces of masonry arch for load combinations.

**Figure 11:**
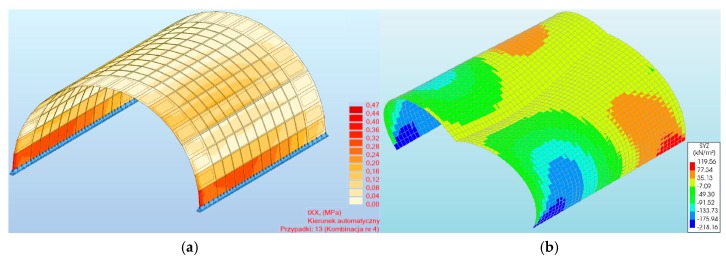
Maximum normal stresses of a three-hinged arch obtained in the program: (**a**) #1, (**b**) #2.

**Figure 12:**
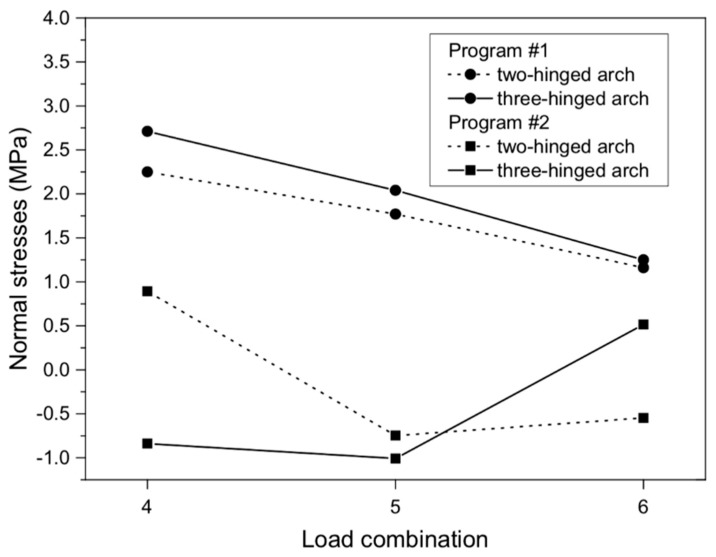
Maximum normal stresses of masonry arch viaduct for various load combinations.

**Figure 13:**
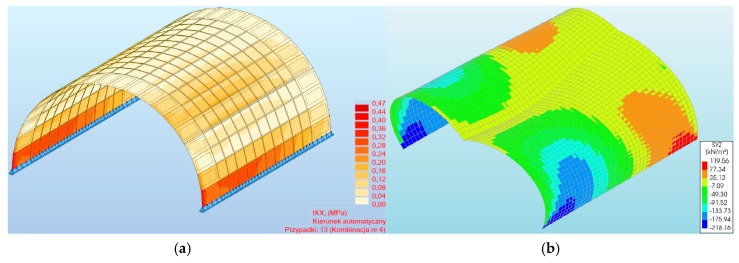
Maximum shear stresses of a three-hinged arch viaduct for the program: (**a**) #1, (**b**) #2.

**Figure 14:**
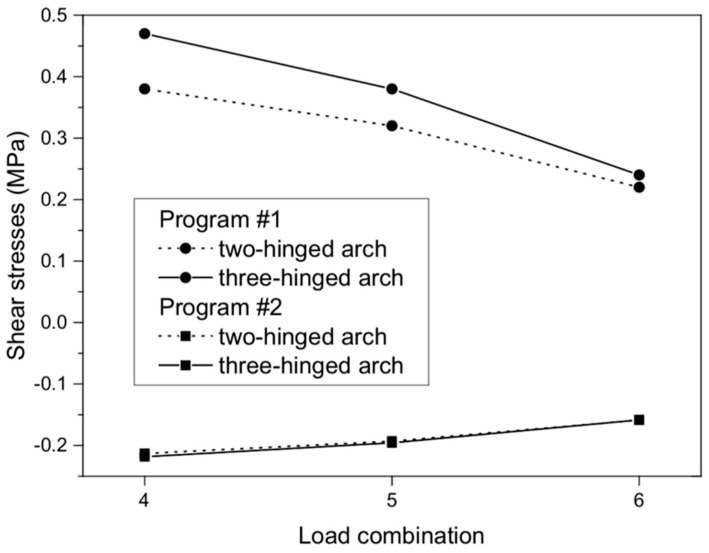
Shear stresses of masonry arch for load combinations.

By contrast, program #2 allows use of a wide spectrum of material models and calculation methods. The limitations in the numerical modelling included using the continuous masonry arch. To best compare the results from the two applied programs, the brick arch was modelled as shell elements. To compare the results from the numerical analysis, three identical load combinations (#4‒6) were selected for each software. The results were presented in the form of maps and their maximum values are shown in Figure 3, Figure 4, Figure 5, Figure 6, Figure 7, Figure 8, Figure 9, Figure 10, Figure 11, Figure 12, Figure 13 and Figure 14 and Table 4.

#### 4.4.2. Results of Numerical Analysis 

For the results from programs #1 and #2, the maximum vertical displacements of the structure at the arch crown (Figure 3a,b) from the standard load were minor and were about 13.10 and 6.7 mm, respectively (for the three-hinged arch). In the case of the two-hinged arch, the maximum displacement at the crown of the viaduct amounted to 7.31 mm. Thus, the largest differences between the maximal displacements of program #1 and #2 did not exceed 47%. Smaller differences were observed in the case of the two-hinged arch model (Figure 4). In all the numerical models, the maximal vertical displacements were observed in the arch crown of the structure.

In the case of bending moments, the maximum values did not exceed 85.46 and 62.64 kNm/m (in the three-hinged arch model) for program #1 and #2, respectively (Figure 5a,b). From Figure 5, for the three-hinged arch, the maximum bending moments were observed in the same places, i.e., between 1/3 and 2/3 of the arch height. In addition, the absolute results in program #1 and #2 for both the two- and three-hinged arch models were relatively close to each other, with the largest differences being 21% and 12%, respectively (Figure 6). In addition, in program #2 for combination #4, the maximum moment was obtained for both the two- and three-hinged bridge models. 

As can be seen in Figure 6, the character of the maximum bending moments in program #2 varied. By contrast, program #1 provided maximum bending moments with a uniform character, i.e., positive. 

When analyzing the axial forces, it was noted that their maximum values (in three-hinged arch) were almost −630 kN/m in program #2 (Figure 7b) and −597.94 kN/m in program #1 (Figure 7a), and these appeared near the foundations of the arch viaduct. Thus, the difference between them was 5%. Meanwhile, for the models with a two-hinged arch, the axial forces were smaller than for the three-hinged arch. For the two-hinged models, the axial forces were −592.10 and −616.15 kN/m for program #1 and #2, respectively. In addition, the results obtained were very similar to each other (Figure 8).

In the case of maximum shear forces, the largest value (−155.00 Kn/m) was also obtained in the numerical model with a three-hinged arch in program #1 (Figure 9a). Using program #2, a 24% lower value was obtained, which was 117.34 Kn/m (Figure 9b). The shear forces obtained in both programs (#1 and #2) appeared around the supports of the numerical models analyzed. In models with a two-hinged arch, shear forces of −127.00 and −109.85 Kn/m were obtained, respectively. Thus, the difference between the programs used for the two-hinged models was 14%. For both axial and shear forces, their character was the same for all the numerical models analyzed (Figure 10). In addition, in programs #1 and #2, the differences between the two- and three-hinged models were 6% and 18%, respectively. 

The maximum normal stresses (2.71 MPa (program #1) and −1.01 MPa (program #2)) were recorded in the three-hinged arch model (Figure 11a,b). These stresses were recorded between 1/3 and 2/3 of the viaduct arch height. Thus, the difference between them was 63%. In addition, a different distribution of normal stresses was noted in both programs for the three-hinged arch model (Figure 12). In program #1, the maximum normal stresses were symmetrical relative to the shell crown (Figure 11a). By contrast, in program #2, it was asymmetrical (Figure 11b). A similar tendency was observed in models with the two-hinged arch, but the maximum normal stress obtained was smaller (Figure 12), i.e., 2.25 (for program #1) and 0.89 MPa (for program #2). One can also observe a good agreement in the results obtained in programs #1 and #2, where the difference between the two- and three-hinged arch viaducts reached 17% and 12%, respectively. 

For shear stresses in the analyzed arch viaduct models, the maximum values were obtained in the three-hinged model and did not exceed 0.47 (program #1, Figure 13a) and –0.22 MPa (program #2, Figure 13b). Thus, the difference between the results of program #1 and #2 was 53%. Shear stresses were observed in the same places, i.e., near the supports of the arch viaduct models. The character of the shear stresses varied (Figure 14). Generally, the differences between the individual arch viaduct models (two- or three-hinged model) in programs #1 and #2 were small (Figure 14) and did not exceed 19% and 5%, respectively (for load combination #4). 

#### 4.4.3. Results Discussion 

The compressive and shear stresses are key to the safety of the masonry arch viaduct. The juxtaposition of the calculated stresses and their comparison with the admissible values (set for the material from which the brick wall of the arch was built) allowed the safety of the viaduct to be verified (Table 5). The compressive and shear strengths of the masonry wall were not exceeded in any case, which proved that the safety of the masonry arch was maintained. The highest effort of the masonry viaduct occurred in a three-hinged arch (load combination (4)). 

The maximal values of compressive and shear stresses were obtained using program #1. In this case, the load-carrying capacity conditions of the masonry arch were satisfied at 84% and 92% due to compressive and shear stresses, respectively. This shows that the load capacity reserves are 16% and 8%, respectively. For other load combinations (5–6), higher load-carrying capacity reserves are obtained (Table 5). In the case of the two-hinged arch, the load-capacity reserve is higher by 8–17% for compression and 9–18% for shearing than those obtained for the three-hinged arch. 

Considering the results received from program #2, the load-carrying capacity reserve is much higher (the static scheme did not have a significant impact on the safety). In the case of a two- and three-hinged arch, the load-capacity reserve was in the range of 68–84% and 57–69% for compressive and shear stresses, respectively. 

The method of masonry arch modelling (simple or advanced) has crucial importance. However, it should be underlined that even simple modelling (with many of the aforementioned limitations) met all load-carrying capacity conditions. 

It should also be mentioned that for almost all the maximum values obtained (displacements, bending moments, axial forces, shear forces, shear stresses), the most unfavorable load case was combination #4 (in both programs).

**Figure 15:**
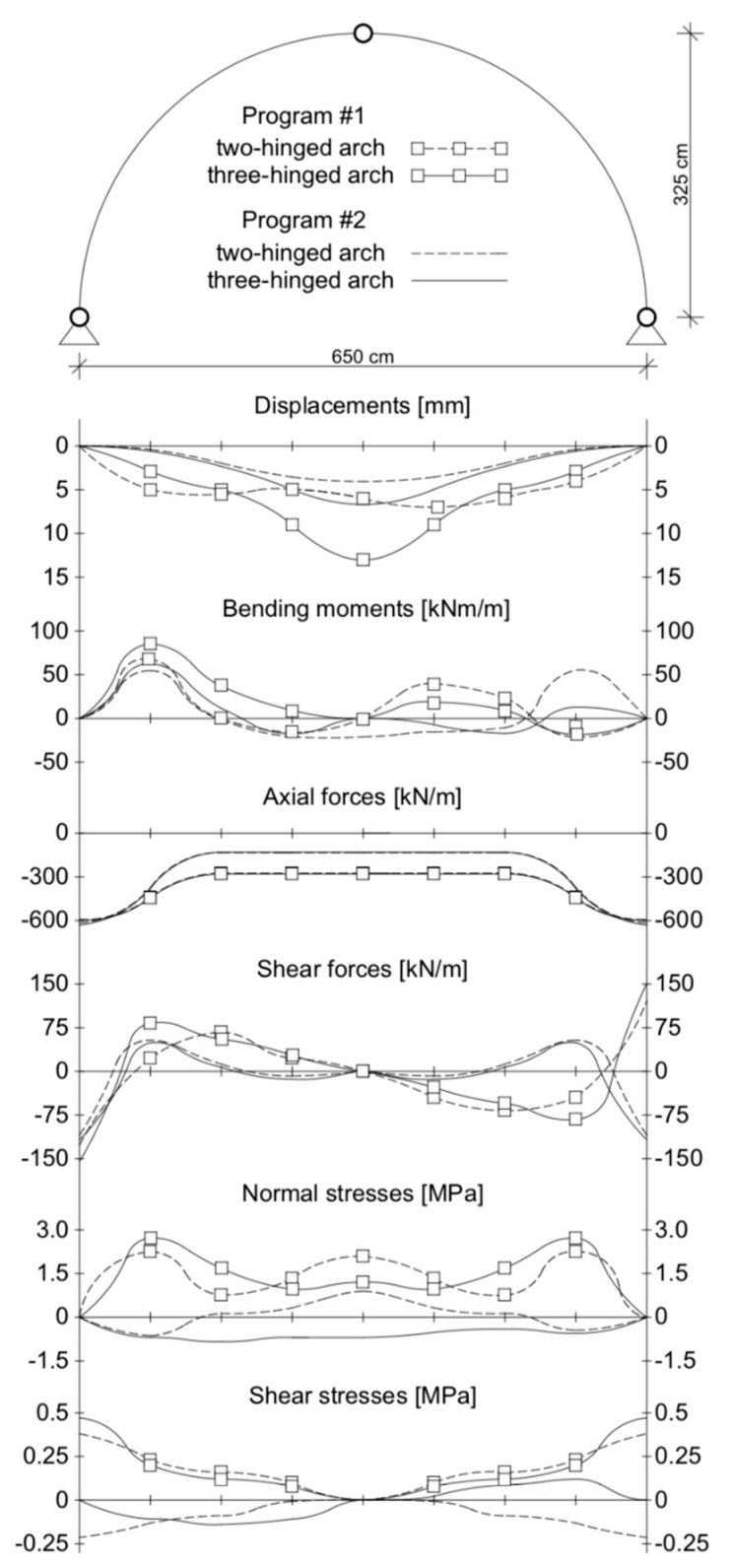
Distribution of analyzed maximum values in the cross-section of the two- and three-hinged arch viaduct in both programs (#1 and #2).

Figure 15 shows the distribution of the analyzed values in the cross-section of the two- and three-hinged arch models. It shows that the maximum displacements were obtained in the arch crown, while the bending moments and normal stresses were at the quarter points of the arch. Meanwhile, the maximum axial forces, shear forces, and shear stresses occurred near the support of the arch viaduct. In almost all cases, the viaduct was symmetrically strained (except for bending moments).

The results from the numerical analysis indicate that the simple model developed in program#1 gives overestimated values compared to the advanced model in program #2. This was due to the fairly simple backfill modelling and the failure to consider the soil–structure interaction in program #1. However, this was a safe approach from a practical point of view. Nevertheless, to obtain reliable information on the actual load-carrying capacity of the viaduct, it is recommended to use the advanced calculation model (program #2).

**Figure 16:**
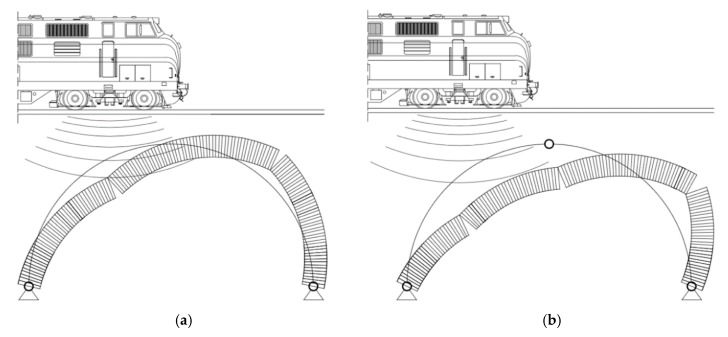
The potential collapse mechanisms for: (**a**) The two- and (**b**) three-hinged arches.

In addition, the potential collapse mechanisms for the two- and three-hinged arches are schematically presented in Figure 16. Potential damage to the arch can occur in the places where the maximum compressive and shear stresses appear. They can appear in the near foundation and quarter points of the arch (for the two-hinged arch), and additionally in the crown for the three-hinged arch. The simplified arch schemes are also presented in [60,61,62], and the influence of failure hinge locations on arch stability is presented in [63]. The authors provided an interactive tool to simplify and aid in understanding the mechanized failure of masonry arches.

## 5. Historic Viaduct Restoration

In relation to the analysis and static-strength calculations performed (there was no risk of loss of carrying capacity of the arch vault), the performance of mostly superficial restoration works has been proposed. Protective works against the ingress of moisture and water into the basic elements of the viaduct have also been planned (vault, front walls, wings). All restoration procedures had to consider the behavior of the original historic character of the masonry viaduct (proposed solutions had to be agreed on with the conservator). 

**Figure 17:**
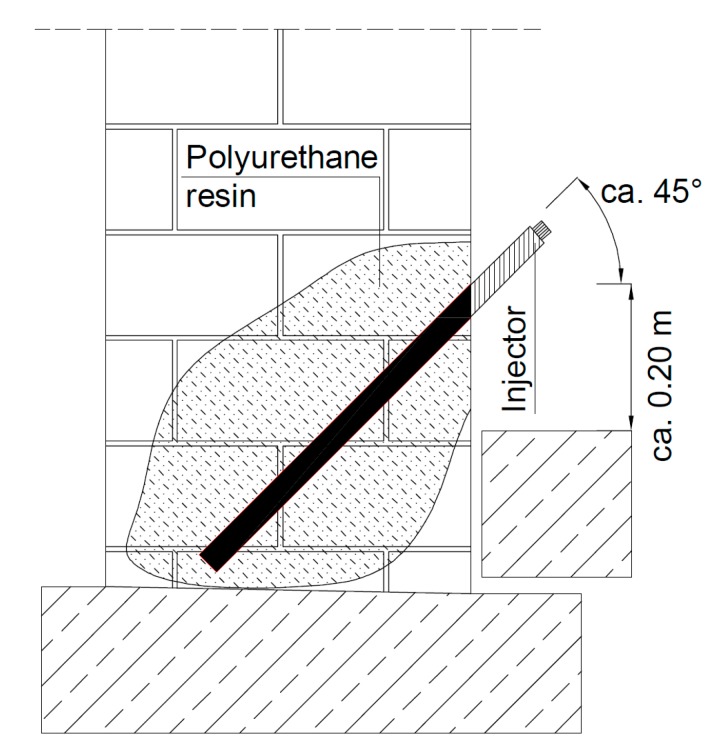
The injection system in the wall.

**Figure 18:**
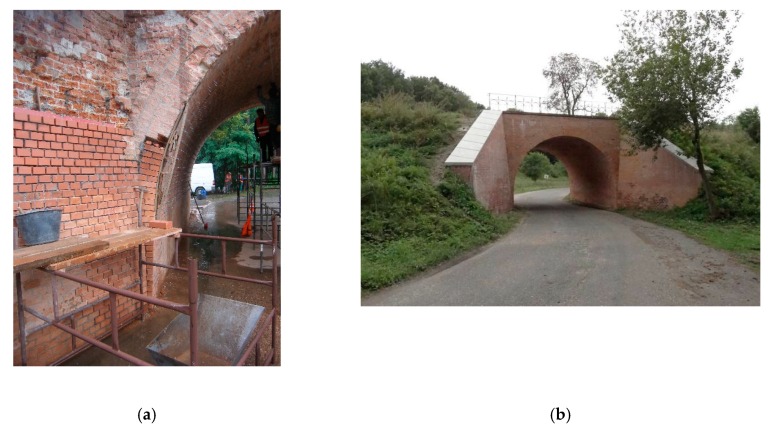
Side view of the masonry viaduct: (**a**) During restoration works (arch injection and rebuilding of the wings), (**b**) after renovation.

Before restoring the viaduct, it was necessary to check the propagation (activity) of cracks using control seals. After a month of viaduct service, none of the control seals was destroyed, which shows that the cracks were stabilized and gaps did not show increases. Restoration and conservation work on the viaduct were performed in the following order:

- All elements of the masonry viaduct (arch, headwalls, parapet, and wings) were thoroughly cleaned from blooms, efflorescence, and stains using a high-pressure cleaner with water. Many loose and crumbling bricks were removed to the depth of all or the shell of a brick. The damaged upper part of the headwall (parapet) was partially rebuilt to the height of the growing root.
- The masonry structure was strengthened using an injection system (Figure 17 and Figure 18a) with the following stages:
  (a) first, holes should be drilled at 45° (Figure 17) and 20 cm spacing (creating a 20 × 20 cm grid with a shift in rows) to a depth of about ¾ of the wall, into which Ø 13/115 mm injectors were placed,
  (b) then, the polyurethane resin had to be injected (characterized by high tensile and flexural strength intended for closing and sealing cracks). Before injection, heavily damaged parts of the wall (mainly joints) had to be sealed with lime-cement mortar, preventing resin from flowing out. After the arch injection, the injectors were removed, and the holes were sealed with the lime-cement mortar.
- After 48 h, the curtain injection waterproofing on the arch and walls began, protecting the wall from exposure to water and moisture. Waterproofing was conducted after strengthening the masonry arch using the injection system. Horizontal insulation of the vault should be made in holes with a 30 × 30 cm spacing drilled through the entire thickness of the walls. A low-viscosity acrylic gel was used to perform the surface insulation.
- Then, losses in brick elements in the headwalls, arch, and wings were supplemented. Joints were made with a special trass mortar with high strength and resistance to water and frost. Facing was performed on all brick parts of the viaduct. Figure 18b shows a view of the masonry viaduct after renovation.
- Then, surface waterproofing (impregnation) of all brick walls was performed using very-low-viscosity epoxy resin. The measure used did not change the external historic character of the viaduct.
- Finally, the area around the viaduct was cleaned up, and drainage pipes were cleaned. New stylized railings with appropriate corrosion protection were installed on the viaduct.

## 6. Conclusions

As a result of the analysis conducted, from calculations and testing the historic railway masonry arch viaduct, the following conclusions have been drawn:

- The masonry arch viaduct showed no tendency to lose stability. It has been proven that for the most unfavorable load conditions, simple viaduct modelling and a static scheme in the form of a three-hinged arch, the safety of the viaduct arch was not exceeded. The possibility of increasing the speed of trains to 40 km/h has been obtained.
- The proposed simple calculation models (as a three-hinged arch) in program #1 allow the safe values of internal forces to be obtained for design purposes. The simple modelling of the arch viaduct in program #1 raises the maximum error compared to program #2, though not by more than 63% (for normal stresses). Additionally, it has been proven that program #2 can yield more reasonable data (the higher load-carrying capacity reserve). However, it requires use of an advanced numerical model including soil-structure interaction phenomena.
- The conditions of the load-carrying capacity of the masonry viaduct arch due to compression and shear are satisfied in all considered cases. The maximum vertical deflections of the masonry arch viaduct were obtained in the arch crown (13.10 and 6.70 mm in program #1 and #2, respectively), while the largest bending moments were located at the quarter points of an arch and did not exceed 86 and 63 kNm/m in program #1 and #2, respectively. The maximum axial forces were observed at support nodes and amounted to –597.94 (program #1) and –630.47 kN/m (program #2). The maximum shearing forces were also located in the supports, from the more loaded side, and amounted to almost –155.00 (program #1) and –117.34 kN/m (program #2).
- The masonry arch was strengthened with polyurethane resin using an injection system. Additionally, both headwalls were rebuilt. Horizontal insulation of the arch with acrylic gel was also applied using curtain injection. Losses in brick elements and mortar in the headwalls, arch, and wings were supplemented. Surface waterproofing (impregnation) of all brick walls was also performed using epoxy resin. The proposed technology for restoring the viaduct will allow safe service for another 25–30 years.

This numerical analysis can be helpful for engineering practice during the reconstruction of masonry arch bridges and may also help designers to create new arch bridges.

## Figures and Tables

**Table 1:** Results of compressive strength for samples of bricks derived from the viaduct arch.

Number of Sample:	Compressive Strength of Brick Sample (MPa)	
	Dried to Constant Mass	Soaked with Water
1	20.62	13.93
2	18.25	15.37
3	23.87	19.12
4	27.00	25.37
5	19.68	11.12
6	15.62	7.93
7	27.56	15.62
8	32.87	20.87
Mean	23.31	16.29
Coefficient of variation (%)	24.64	34.27

**Table 2:** Compressive and shear strength of the brick wall part derived from the viaduct arch.

Number of Sample:	Compressive Strength (MPa)		Shear Strength (MPa)
	in lab	in-situ	in lab
1	14.8	14.8	0.40
2	12.9	21.9	0.38
3	18.2	15.0	0.37
4	16.5	15.8	0.39
5	16.1	17.6	0.37
6	15.2	18.7	0.38
7	15.5	17.3	0.36
Mean	15.6	17.3	0.378 ≈ 0.38
Coefficient of variation (%)	10.44	14.35	3.55

**Table 3:** Additional load combinations applied for the analyzed masonry arch viaduct.

# load Combinations	Load Description
(1)	A, B, C, D, E
(2)	A, 1/2B, C, D, E
(3)	A, 1/4B, C, D, E
(4)	A, B, C, D, E, F
(5)	A, 1/2B, C, D, E, F
(6)	A, 1/4B, C, D, E, F

Note: A – soil weight and ballast, B – load with rolling stock, C – load with braking and acceleration, D – side-impacts, E – dead weight of the arch, F – active earth pressure.

**Table 4:** Juxtaposition of maximum internal forces in the masonry viaduct (three- and two-hinged arch) for the applied load combinations.

	Program #1		Program #2	
# Load Combination	Two-Hinged Arch	Three-Hinged Arch	Two-Hinged Arch	Three-Hinged Arch
Displacements (mm)				
(4)	6.51	13.10	4.06	6.70
(5)	7.31	8.91	3.61	3.99
(6)	3.77	4.23	2.41	2.42
Bending moments (kNm/m)				
(4)	67.68	85.46	55.24	62.64
(5)	58.10	69.99	–50.47	47.34
(6)	35.19	37.60	–48.98	–51.83
Axial forces (kN/m)				
(4)	–592.10	–597.94	–616.15	–630.47
(5)	–520.77	–530.28	–494.27	–499.83
(6)	–394.80	–396.17	–387.05	–380.33
Shear forces (kN/m)				
(4)	–127.00	–155.00	–109.85	–117.34
(5)	–107.74	–125.61	–82.49	–86.16
(6)	–72.40	–79.56	–63.24	–61.82
Normal stresses (MPa)				
(4)	2.25	2.71	0.89	–0.84
(5)	1.77	2.04	–0.75	–1.01
(6)	1.16	1.25	–0.55	0.51
Shear stresses (MPa)				
(4)	0.38	0.47	–0.21	–0.22
(5)	0.32	0.38	–0.19	–0.20
(6)	0.22	0.24	–0.16	–0.16

**Table 5:** Masonry arch safety for the applied load combinations and models used.

	Program #1		Program #2	
# Load Combination	Two-Hinged Arch	Three-Hinged Arch	Two-Hinged Arch	Three-Hinged Arch
Arch effort due to compressive stresses (*σ*_c, FEM_ / *f*_d,c_) × 100%				
(4)	70	84	27	26
(5)	55	63	23	32
(6)	36	39	17	16
Arch effort due to shear stresses (*τ*_s, FEM_ / *f*_d,s_) × 100%				
(4)	75	92	41	43
(5)	63	75	37	39
(6)	43	47	31	31

Note: σ_c, FEM_ and τ_s, FEM_ – compressive and shear stresses obtained from the numerical analysis, respectively. *f*_d,c,_ and *f*_d,s_ – design compressive (3.20 MPa) and shear (0.51 MPa) strength of the brick wall, respectively.
